# High Intensity Interval Training Leads to Greater Improvements in Acute Heart Rate Recovery and Anaerobic Power as High Volume Low Intensity Training

**DOI:** 10.3389/fphys.2017.00562

**Published:** 2017-08-02

**Authors:** Thomas L. Stöggl, Glenn Björklund

**Affiliations:** ^1^Department of Sport Science and Kinesiology, University of Salzburg Salzburg, Austria; ^2^Department of Health Sciences, Sports Tech Research Centre, Mid Sweden University Östersund, Sweden; ^3^The Swedish Sports Confederation Stockholm, Sweden

**Keywords:** lactate threshold, peak power, maximal anaerobic running test, mart, MACT, training intensity distribution

## Abstract

The purpose of the current study was to explore if training regimes utilizing diverse training intensity distributions result in different responses on neuromuscular status, anaerobic capacity/power and acute heart rate recovery (HRR) in well-trained endurance athletes.

**Methods:** Thirty-six male (*n* = 33) and female (*n* = 3) runners, cyclists, triathletes and cross-country skiers [peak oxygen uptake: (VO_2peak_): 61.9 ± 8.0 mL·kg^−1^·min^−1^] were randomly assigned to one of three groups (blocked high intensity interval training HIIT; polarized training POL; high volume low intensity oriented control group CG/HVLIT applying no HIIT). A maximal anaerobic running/cycling test (MART/MACT) was performed prior to and following a 9-week training period.

**Results:** Only the HIIT group achieved improvements in peak power/velocity (+6.4%, *P* < 0.001) and peak lactate (*P* = 0.001) during the MART/MACT, while, unexpectedly, in none of the groups the performance at the established lactate concentrations (4, 6, 10 mmol·L^−1^) was changed (*P* > 0.05). Acute HRR was improved in HIIT (11.2%, *P* = 0.002) and POL (7.9%, *P* = 0.023) with no change in the HVLIT oriented control group.

**Conclusion:** Only a training regime that includes a significant amount of HIIT improves the neuromuscular status, anaerobic power and the acute HRR in well-trained endurance athletes. A training regime that followed more a low and moderate intensity oriented model (CG/HVLIT) had no effect on any performance or HRR outcomes.

## Introduction

Endurance athletes use different training strategies to improve their performance. Although the bulk of training sessions typically are made up of longer and at slower paced session's (Tonnessen et al., 2014), intervals and higher exercise intensity sessions are a necessity for high performance. To implement diverse types of exercise intensities and durations, athletes use various ways of periodization in their training. The type of periodization depends on the sport and the length of the competition season which decides the duration of the training period. The most common ways for periodization is (i) high volume low-intensity training (HVLIT), lactate threshold training (THR), low-volume high-intensity (interval) training (HIIT) and polarized training, a concept consisting of mixing training between low and high intensity (POL) or a gradual decrease in training volume from HVLIT to THR and HIIT in a pyramidal fashion (Stöggl and Sperlich, 2015)

Athletes use various tests for evaluation of the training process to accurately target specific elements that are important for performance. Depending on the sport, different quality's such as cardiovascular, muscular, or metabolic variables are key elements for performance to a various degree. The most common way for endurance athletes to track and overview their daily training is the use of heart rate (HR) monitoring (Achten and Jeukendrup, 2003). One of the most significant cardiovascular enhancements is an increased stroke volume which is associated with a lower exercise HR for a given submaximal work (Blomqvist and Saltin, 1983). Furthermore, HR recovery (HRR) after cessation of exercise has been put forward as a useful indicator to track cardiovascular advances for athletes of various levels (Daanen et al., 2012). As an example, HRR was tracked in well-trained cyclists throughout a training period of 4 weeks constituting 8 × 4 min HIIT two times per week (Lamberts et al., 2009). It was concluded that well-trained athletes, who responded well to this type of training, demonstrated a faster HRR after the interval session and after a 40-km time trial (HR drop during the 60 s post-exercise) that further was related to an enhanced endurance performance. To note, that no other training- or control-group was included in this study. Additionally, HRR relating to both the drop and the time to reach a certain beat per minute, responds differently to various forms of interval training regimes (Buchheit et al., 2008). This indicates that HRR could provide beneficial feedback for cardiovascular adjustments, not only during exercise, but also in between exercise bouts.

Another frequent used tool for determination of physiological response is determination of blood lactate concentration. Most often blood lactate is related to an increase in exercise intensity to identify various lactate thresholds (Beneke et al., 2011). During variable intensity exercise blood lactate recovery has been suggested as a good indicator for performance both for cycling and cross-country skiing (Björklund et al., 2007, 2011). These latter studies were conducted on well-trained athletes. Also, different types of training seem to stimulate the lactate removal abilities as middle distance runners surpass sprint runners regarding lactate recovery in between high intensity bouts (Bret et al., 2003).

Performance tests with intermittent character are a common and valid instrument to explain performance progress that do not relate to aerobic characteristics. For evaluation of the athletes' neuromuscular status and anaerobic power maximal anaerobic treadmill tests are a useful assessment, i.e., MART (Paavolainen et al., 1999b; Nummela et al., 2006). The test is of intermittent character with an increase in exercise intensity for consecutive bouts and is terminated at volitional fatigue. This test relates to the individuals anaerobic input to the exercise performance and could therefore provide an estimate which areas the athletes lack and need to improve for enhanced performance. To note here, that the effects of different endurance training concepts on key components of endurance performance were shown previously (Stöggl and Sperlich, 2014), while the effects of these training concepts on anaerobic power and HRR are lacking.

The aim of the study was to evaluate the effects of different training concepts (POL vs. HIIT vs. HVLIT) with respect to anaerobic power, cardiovascular and metabolic response using key measurements during the MART and HRR. We hypothesized that athletes who use training concepts involving high intensity elements, i.e., HIIT and POL, would display superior improvements compared with athletes that use no HIIT (i.e., HVLIT).

## Materials and methods

### Participants

Thirty-six competitive endurance athletes (three females and 33 males) who participated in either cross-country skiing, cycling, triathlon, middle- or long-distance running volunteered to take part in this study (mean ± *SD*: age: 31 ± 6 yrs, body mass: 74.6 ± 8.9 kg, height: 180 ± 7 cm) were recruited from regional cycling, running, triathlon, athletic, and cross-country skiing clubs. All participants were well-trained athletes [61.9 ± 8.0 mL·kg^−1^·min^−1^ (range: 54–75 mL·kg^−1^·min^−1^)], accustomed to a training frequency of more than five sessions per week (totally 10–20 h·wk^−1^), participated frequently in endurance competitions for the last 8–20 years and were healthy throughout the intervention period. Participants were members or former members of the Austrian cross-country skiing national team (*n* = 8), runners and triathletes (*n* = 10) or cyclists (*n* = 13) of regional sport teams during or since the year before the current study. Retrospective analysis of the 6 months training prior to the study revealed that none of the participants had regularly engaged HIIT. Instead all had used a HVLIT training protocol with a maximum of two THR training sessions per week.

Based on the participants' baseline VO_2max_ and training mode (running or cycling), all athletes were parallelized into three groups: HIIT, POL, and control group (CG; HVLIT oriented with 1–2 THR sessions per week). At baseline, the three groups were not statistically different with regard to age, height, body mass, or VO_2max_. During an initial visit, study details, and participation requirements were explained, and all participants gave written informed consent. The study and protocol received approval from the University of Salzburg Austria Ethics Committee and was conducted in accordance with the Declaration of Helsinki.

### Design

The intervention lasted 9 weeks plus 2 days of pre- and post-testing. All athletes who were mainly engaged in cycling training during the intervention period trained with their own bike and completed all tests on a bicycle ergometer (Ergoline, Ergoselect 100P; Bitz, Germany) using their own cycling shoes and pedal system. Other athletes ran during the study and completed their pre- and post-testing on a motorized treadmill (HP Cosmos, Saturn, Traunstein, Germany). All participants were instructed not to change their diet throughout the training period and to maintain strength training, if it was part of their training program. Participants' nutritional intake was not standardized or controlled during the study, but for the 3 h prior to all testing in which food intake was not permitted. The training intensity was controlled by HR based on the baseline incremental test: (i) low intensity training (LIT, HR at blood lactate value <2 mmol·L^−1^); (ii) moderate intensity training (MIT, HR corresponding to a blood lactate of 3–5 mmol·L^−1^); (iii) high intensity interval training (HIIT, >90% HR_max_)] (e.g., Seiler, 2010; Stöggl and Sperlich, 2015). The HR was measured during each training session and athletes documented training mode, exercise duration and intensity in a diary. As a control and for detailed analysis, HR for all training sessions was stored digitally and analyzed retrospectively. For the quantification of the training intensity distribution within the 9-weeks of training the session goal approach according to Seiler and Kjerland (2006) was applied.

### HIIT intervention

The HIIT included two interval blocks of 16 days with one adaptation week prior to and one recovery week after each block. The adaptation week included two 60 min HIIT sessions, three 90 min LIT sessions, one 120 min LIT session and 1 day of recovery. The condensed 16 day interval block included 12 HIIT sessions within 15 days, integrating four blocks of three HIIT sessions for 3 consecutive days followed by 1 day of recovery. The recovery week contained four LIT sessions of 90 min and 3 days without any training. All of the HIIT sessions included a 20 min warm-up at 75% of HR_max_, 4 × 4 min at 90–95% of HR_max_ with 3 min active recovery and a 15 min cool-down at 75% HR_max_ based on the protocol proposed earlier (Helgerud et al., 2007). The LIT sessions lasted 90–150 min depending on the training mode (running vs. cycling) at an intensity resulting blood lactate of <2 mmol·L^−1^.

### POL intervention

The POL included three blocks, each lasting 3 weeks: 2 weeks of high volume and intensity training followed by 1 week of recovery. The high volume and intensity week included six sessions with two 60 min HIIT sessions, two 150–240 min long duration LIT sessions (duration according to training mode: cycling, running or roller skiing), which included six to eight maximal sprints of 5 s separated by at least 20 min, and two 90 min LIT sessions. The recovery week included one 60 min HIIT session, one 120–180 min LIT session and one 90 min LIT session.

### Control group (CG/HVLIT)

The CG continued their HVLIT dominated training regime with a maximum of two THR sessions per week with no HIIT sessions. The control group also had three blocks each lasting 3 weeks with 2 weeks of high-volume training followed by 1 week of recovery.

### Pre- and post-testing

All participants were asked to report well-hydrated and to refrain from consuming alcohol and caffeine for at least 24-h, as well as from engaging in strenuous exercise at least 48-h prior to testing. The pre- and post-tests included a VO_2max_ ramp protocol and a maximal anaerobic running/cycling test (MART/MACT) based on the protocol of Rusko et al. (1993) in running and Tossavainen et al. (1996) in cycling.

On the first test day all athletes completed a VO_2max_ ramp protocol to determine maximal oxygen uptake (VO_2max_) and maximal HR (HR_max_). First, the workload for running was set at 8 km·h^−1^ (inclination: 5%) on the treadmill, and for cycling at 200 W with a cadence of >80 rpm for 10 min. The workload was then increased every 30 s by 0.5 km·h^−1^ (inclination: 10%) on the treadmill or 15 W on the cycle ergometer until exhaustion. VO_2_ was measured with an open circuit breath-by-breath spirograph (nSpire, Zan 600 USB, Oberthulba, Germany), which was calibrated prior to each test using high precision gas (15.8% O_2_, 5% O_2_ in N; Praxair, Düsseldorf, Germany) and a 1 L syringe (nSpire, Oberthulba, Germany). All respiratory data were averaged every 30 s.

**Figure 1:**
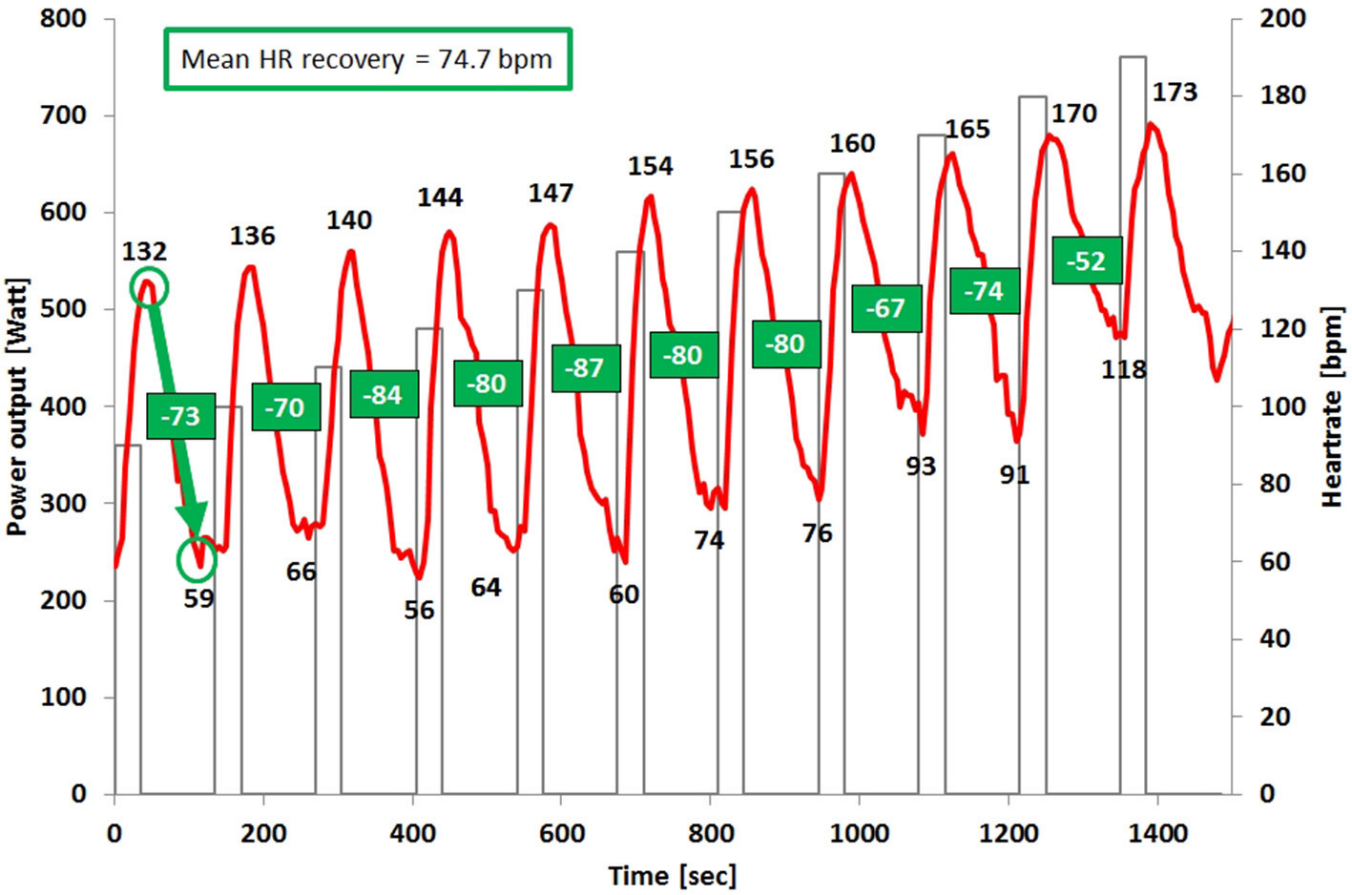
Illustration of the heart rate-time curve and the heart rate recovery calculation within the MART/MACT of one subject.

On the second day athletes performed the MART/MACT. The protocol included stages of 25 s (running) or 30 s (cycling; including 3–5 s acceleration time) with 100 s breaks in between. For the running protocol treadmill speed was increased with 1.4 km·h^−1^ increments starting at 14.7 km·h^−1^ on a grade of 7%. For the cycling protocol the test started at 360 W with increments of 40 W. Maximal performance (V_max_) in the MART was calculated by linear interpolation using the formula: V_max_ = V_*f*_ + ((t/25) 1.4 km·h^−1^), where V_*f*_ was the velocity of the last completed workload (km·h^−1^), t the duration of the last workload (s) and 1.4 m·s^−1^ the velocity difference (ΔV) between the last two workloads. For the MACT, the formula for maximal power output (P_max_) was: P_max_ = P_*f*_ + ((t/30) · 40 W), with P_*f*_ as the power output of the last completed stage. A 20 μl blood sample from the right earlobe was collected within the 60 s of each 100 s rest period, and in the first, third, fifth and seventh minutes after the end of the last stage into a capillary tube (Eppendorf AG, Hamburg, Germany). All samples were analyzed amperometric-enzymatically (Biosen 5140, EKF-diagnostic GmbH, Magdeburg, Germany) in duplicate, and the mean of the two measures was used for statistical analysis. The lactate sensor was calibrated before each test using a lactate standard sample of 12 mmol·L^−1^. Results within a range of ±0.1 mmol·L^−1^ were accepted. Velocity/power output at 4, 6, and 10 mmol·L^−1^ of blood lactate were calculated. HR recovery (HRR) was calculated as the mean value of all delta changes of each stages peak HR (highest value at the end or in the first seconds after the end of the stage) and minimal HR (minimum value during the 100 s break; Figure 1).

### Statistical analyses

All data exhibited a Gaussian distribution verified by the Shapiro–Wilk's test and, accordingly, the values are presented as means ± *SD*. Two-way 2 × 3 repeated-measures ANOVA (2 times: pre-post, 3 groups) to test for main effects of time (pre- and post-intervention), group (the three training groups) and the interaction effect between both factors was applied. When a significant main effect over time and/or interaction effect was observed, paired *t*-tests within each group were conducted. Based on the different units of peak velocity/power in the MART/MACT percent changes between pre- to post-values were calculated, and a one-way ANOVA between groups was performed using Tukey's *post-hoc* analysis. Furthermore, within group changes for these variables were calculated using Wilcoxon tests. In addition, the values obtained were evaluated further by calculating the effect size (_p_η^2^). The magnitude of the difference was classified as trivial (<0.01), small (0.01 ≤ to < 0.06), moderate (0.06 ≤ to < 0.14) or large (≥0.14). An alpha value of <0.05 was considered significant. The Statistical Package for the Social Sciences (Version 24.0; SPSS Inc., Chicago, IL, USA) and Office Excel 2010 (Microsoft Corporation, Redmond, WA, USA) were used for statistical analysis.

## Results

Thirty-one participants completed the 9-week training protocol, fulfilling more than 95% of the training program and staying within the given HR zones. Seven subjects (2 in HIIT and 4 in CG) withdrew from the study due to illness (*n* = 2) or were excluded due to changes in competition schedule (*n* = 2). The total training hours, number of training sessions and their percent distribution within LIT, MIT, and HIIT are presented in Table 1. POL and CG/HVLIT had higher training volume (*P* < 0.001) and number of trainings session (*P* = 0.041) compared with HIIT. The training intensity distributions with respect to LIT, MIT, and HIIT were 68/6/26% for POL, 43/0/57% for HIIT and 64/35/1% for CG/HVLIT. HIIT demonstrated the lowest number of LIT sessions and CG/HVLIT the highest number of MIT sessions with no difference between the two other groups. HIIT sessions were greatest in HIIT followed by POL and finally CG/HVLIT.

**Table 1:** Volume and intensity training distribution within the 9-weeks training intervention (excluding strength training).

	**POL**	**HIIT**	**CG/HVLIT**	***P*-value**
Total hours	104 ± 21	66 ± 1^*^	93 ± 13	<0.001
Number of sessions	54 ± 7	47 ± 1^§^	54 ± 8	=0.041
Number of LIT training sessions	37 ± 9	20 ± 1^*^	36 ± 15	=0.004
Number of MIT training sessions	3 ± 4	0 ± 0	18 ± 9^*^	<0.001
Number of HIIT training sessions	14 ± 3^*^	27 ± 1^*^	0 ± 1^*^	<0.001
Percent LIT training sessions	68 ± 12%	43 ± 1%^*^	64 ± 20%	=0.002
Percent MIT training sessions	6 ± 7%	0 ± 0%	35 ± 21%^*^	<0.001
Percent HIIT training sessions	26 ± 7%^*^	57 ± 1%^*^	1 ± 1%^*^	<0.001

*The values presented are means ± SD. P-values were obtained by one-way ANOVA (3 training groups). POL, polarized training group; HIIT, High intensity interval training group; CG /HVLIT, control group with mainly high volume low intensity training; LIT, low intensity training; MIT, moderate intensity training; HIIT, high intensity interval training*.

* *Different from all other groups*.

§ Different from training group “CG/HVLIT.”

Percent changes in variables from pre- to post-training and between the training concepts during the MART/MACT are presented in Table 2. For P/V_peak_ there was a main effect of time and interaction effect time × group (both *P* = 0.001) with HIIT demonstrating the greatest increase (6.4 ± 3.4%, *P* < 0.001) with no significant change in POL (0.2 ± 5.9%, *P* = 0.63) and CG/HVLIT (4.7 ± 5.5%, *P* = 0.087).

**Table 2:** Per cent changes in velocity (V) and power (P) and at various lactate thresholds as well as peak velocity and power.

	**POL**	**HIIT**	**CG/HVLIT**	***P*-value, Effect size _p_η^2^**
V/P 4 (%)	−1.6 ± 13.1	4.1 ± 9.6	3.2 ± 13.04	NS, 0.03
V/P 6 (%)	3.3 ± 13.8	1.8 ± 6.5	1.1 ± 8.3	NS, 0.03
V/P 10 (%)	2.8 ± 9.6	0.1 ± 5.5	2.7 ± 7.5	NS, 0.03
V/P_peak_ (%)	0.2 ± 5.9^†^	6.4 ± 3.4^***^	4.7 ± 5.5	= 0.033, 0.22
LA_peak_ (%)	−6.6 ± 13.3^†^	7.3 ± 4.7^***^	1.3 ± 12.3	= 0.030, 0.22
HR_peak_ (%)	−0.8 ± 3.9	−0.5 ± 2.8	0.0 ± 3.1	NS, 0.01
HRR (%)	7.9 ± 9.7^*^	11.2 ± 7.7^**^	0.1 ± 5.6^‡^	= 0.011, 0.28

The values presented are means ± SD. P-values were obtained by one-way ANOVA (three training groups) calculated over the per cent differences between pre- to post-training (representing the interaction effect time × group). POL, polarized training group; HIIT, High intensity interval training group; CG/HVLIT, control group with focus on high volume training; V/P4 mmol·L^-1^, velocity or power at 4 mmol·L^-1^ blood lactate; V/P6 mmol·L^-1^, velocity or power at 6 mmol·L^-1^ blood lactate; V/P10 mmol·L^-1^, velocity or power at 10 mmol·L^-1^ blood lactate; V/P_peak_, peak velocity or power in the MART/MACT; LA_peak_, peak lactate during the test and within the first 7 min after end of the last completed stage; HR_peak_, peak heart rate value during the MART/MACT; HRR, mean heart rate recovery;

* *p < 0.05*,

** *p < 0.01*,

*** *p < 0.001 significant difference within groups from pre- to post-training*.

† *Significant different from HIIT group*.

‡ *Significant different to both other groups. NS, not significant*.

**Figure 2:**
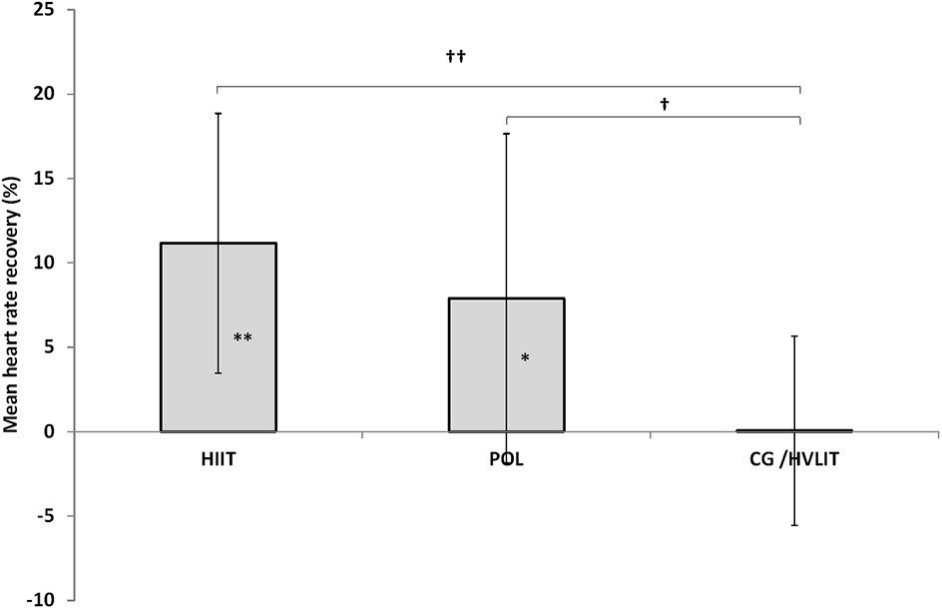
Per cent changes of mean heart rate recovery within the three training groups between pre- and post-intervention testing. ^*^p < 0.05, ^**^p < 0.01 significant difference within groups from pre- to post-training. ^†^*p* < 0.05, ^††^*p* < 0.01 significant different to CG/HVLIT group.

For HRR there was a main effect of time (*P* < 0.001) and interaction effect time × group (*P* = 0.011) with HIIT (38.7 ± 10.7 to 49.9 ± 14.1 bpm, 11.2%, *P* = 0.002) and POL (48.9 ± 15.9 to 56.8 ± 22.0 bpm, 7.9%, *P* = 0.023) demonstrating greater increases compared with unchanged levels of 0.1% in CG/HVLIT (49.3 ± 7.5 to 49.4 ± 9.3 bpm, *P* > 0.05) (Figure 2). All significant main and interaction effects demonstrated large effect sizes (>0.14).

LA_peak_ demonstrated a time × group interaction effect (*P* = 0.027) with a 7.3% (*P* = 0.001) increase in HIIT and non-significantly changed values of −6.6% in POL and +1.3% in CG/HVLIT (both, *P* > 0.05).

No changes from pre to post and no differences between training groups were detected with respect to HR_peak_ and velocity /power at 4, 6, and 10 mmol·L^−1^ blood lactate (all *P* > 0.05).

## Discussion

The major findings of the study were that (i) only the HIIT group improved their peak velocity or power output in the MART/MACT, (ii) HRR was faster in the HIIT and POL groups compared with no change in the CG/HVLIT group, (iii) while no training intervention improved the velocity or power output at the established lactate concentrations during the MART/MACT.

### Anaerobic power

One of the major findings of this training study were the enhanced P/V_peak_ for the HIIT group, demonstrating the greatest increase (+6.4%), with no notable change in POL and CG/HVLIT. Earlier data using cross-sectional comparisons between different types of athletes in running (Nummela et al., 1996) or cross-country skiing (Stöggl and Müller, 2009) showed that P/V_max_ of the MART was determined by metabolic variables as peak lactate and power output or velocity at 10 mmol·L^−1^. The importance of these variables and especially the velocity at 10 mmol·L^−1^ have further been strengthened by the same research group (Nummela et al., 2007). While this cross-sectional data showed important features for anaerobic power reflected in the MART, there was no quantification of the athletes training that preceded the test. In the current study, the HIIT group was the exclusive training modality that had a positive effect on the MART/MACT performance. Indeed, the peak lactate and P/V_peak_ was increased in the HIIT group while it remained unchanged in the other two groups (POL and CG/HVLIT). Certainly, a greater glycolytic activity, which involves formation of lactate, is favorable to produce ATP at a higher rate and likewise should add to the overall performance in the MART/MACT. Therefore, the HIIT intervention seems to have substantial impact on both the metabolic and neuromuscular components of maximal anaerobic performance.

The relationship between the MART and the maximal anaerobic oxygen deficit (MAOD) as well as the energy contribution during the MART was already investigated by Zagatto et al. (2011). Although, the relationship between the MART and MAOD was poor, the quantification of the energetic contribution demonstrated that the aerobic input covers the greatest amount of energy production during the full test, i.e., including both the work and rest periods (65%), while the anaerobic glycolytic energy system contributed with approximately only 5%. When analyzing only the effort periods (25 s) the anaerobic contribution corresponded to ~74% with the main energy system being the a-lactic (63%) and not the glycolytic lactic system (11%). Moreover, it has been shown that the aerobic contribution increases already at the second repeated bout in sprint exercise (Bogdanis et al., 1996). Therefore, the relation to peak lactate concentration and performance in the MART/MACT within the current study seems conflicting.

The neuromuscular capacity to produce force was shown to be related to both the MART and 5-k running performance (Nummela et al., 2006). Furthermore, close relationships between running performance from distances of 400–5,000 m with performance in the MART and short-duration sprint performance over 20–30 m were found in numerous studies (e.g., Rusko et al., 1993; Nummela et al., 1996, 2006, 2007; Paavolainen et al., 1999a,c). In this context, neuromuscular adaptations using HIIT in ice hockey players, improved the general muscle activation as demonstrated in increased force and rate of force development in an isolated plantar flexion (Kinnunen et al., 2017). Shortcomings in the study of Kinnunen et al. (2017) were the missing transfer of the results to increased sport specific performance (e.g., faster skating times on ice). Compared to the current study, in the study by Kinnunen et al. (2017) a shorter training period (2.5 vs. 9 weeks) and shorter HIIT bouts (30 s vs. 4 min) were applied. The question remains which training period durations using different training regimes (e.g., HIIT protocols) are necessary for these improvements. While these shorter HIIT bouts relate more to team sports as ice hockey, there seem to be also positive effects for sprint triathlon performance where subjects used a mix of short and long bouts (Garcia-Pinillos et al., 2017). In summary, different HIIT regimes have been proven to have a significant impact on trained endurance athletes for endurance performance (Laursen et al., 2002). In all, the improved MART/MACT performance in the current study potentially translates to an improved performance in the athlete's specific sport.

### Heart rate recovery (HRR)

The two training groups including high intensity sessions, HIIT and POL, displayed a superior HRR during the MART/MACT compared with CG/HVLIT. In detail, HIIT demonstrated the greatest per cent change in HRR (11.2%) followed by POL (7.9%) and no change in CG/HVLIT (0.9%). Conventionally, HRR is measured within the first 60 s after termination of a test or training (Lamberts et al., 2009). In this study, the HRR represents the mean of the acute HRR in between several stages during the MART/MACT. This is the first time that data during the specific MART/MACT has been shown. HRR was found to be different between trained and untrained healthy individuals and that improvements in HRR occur with an increase in training status (Daanen et al., 2012). Also this improved HRR has been used as a reliable test that relates to various performances as longer time-trials as well as peak power output (Lamberts et al., 2011). However, there are conflicting results showing a decreased performance for intermittent high intensity exercise in athletes that show an enhanced HRR (Le Meur et al., 2016). Furthermore, it was suggested that an improved HRR could be biased by a decreased maximal HR. None of the groups in the current study displayed such a pattern as all of them maintained their maximal HR. Even though, it is well-established that the stroke volume increases as a result of endurance training (Blomqvist and Saltin, 1983) the reason for the improved HRR is related rather to the nervous system. A delayed parasympathetic reactivation has been proposed to be part of the HRR post-exercise (Buchheit et al., 2007). Interestingly, the HRR seems to be acutely impaired by a high anaerobic contribution. However, the question is if this is trainable and might be different if the athlete is accustomed to more anaerobic work. Our results indicate that the athletes exposed to HIIT seemed to handle the anaerobic stress better than the HVLIT dominated groups indicated by their superior HRR. Differences in training load can impact HRR (Borresen and Lambert, 2007) as demonstrated by an attenuated HRR following greater training load, defined according to the TRIMP method. In the current study, the HIIT had a markedly lower training load when compared to all other modalities when counting training hours (<70 vs. ~100 h). Nevertheless, even though the training load was not calculated according to the TRIMP method, the HIIT clearly showed a lower training load based on duration and frequency. Contradictory to our results, regarding training hours, it has been shown that severe increase in training hours per week markedly increases HRR along with a concomitant loss of performance of a single time-trial (Thomson et al., 2016). Notably in our study, the increase in training hours was accompanied with an increase in percent of high intensity exercise which makes it difficult to pin point if it is hours or high intensity exercise that sole alone explain the outcome.

Another aspect for the more pronounced HRR in the groups including high intensity sessions (HIIT and POL) might be the intermittent character of HIIT itself. Possibly, the repeated steady change of high and low intensities within the sessions might be an appropriate stimulus to enhance the ability of the autonomic nervous system to acutely adapt toward changing intensities. Future studies would be of a necessity to cover this area to explore the exact mechanisms.

In all, the training groups that included HIIT (POL and HIIT) both showed an increased HRR. While an enhanced HRR has been interpreted to be part of a functional overreaching with a decreased performance, the short tapering period (days) in the current study has quickly affected the performance in a positive direction. Therefore, the HIIT group likely had sufficient time for recovery in between training sessions to show both improvements in HRR concomitant with performance.

### Velocity or power output at absolute lactate concentrations

In the current study, neither the velocity nor the power output at any of the established lactate concentrations (4, 6, and 10 mmol·L^−1^) showed any improvements. More specific, it is interesting that the CG/HVLIT as the only training group that targeted training at the defined lactate concentration (e.g., approximately two sessions of THR/week) lacked any development in velocity or power output. Interestingly, the lack of improvement is somewhat unexpected as the especially enhanced performance in running is explained by a right shift for lactate threshold in relation to velocity (vLT; Billat et al., 2002). Notably, this change was apparent already after a 4-week intervention.

Another study using HIIT and more traditional training as long slow distance (LSD) displayed increased power outputs for both training modalities at 2 and 4 mmol·L^−1^ but with superior development in the HIIT group (Ni Cheilleachair et al., 2017). Their study resembled the adaptation period of the current study as they used an 8-week training intervention. The use of rowers as subjects could likewise be compared to the cross-country skiers in the current study as both sports use whole body work for propulsion. In support of these data a study performed on cyclists comparing HIIT block periodization with a more traditional training regime, i.e., mostly low intensity sessions with a few HIIT sessions (Ronnestad et al., 2014), showed that only the HIIT block periodization increased power output at 2 mmol·L^−1^. The training performed was rather similar to the current study using HIIT session of target HR at 88–100% of HR_max_. The approximately accumulated time at this exercise intensity was 30 min for each occasion in their study. It might be that the stimulus that was used in the current study was too short in duration as it contained 16 min in total per session. However, we used a longer training period that spanned more than twice the weeks (4 vs. 9 weeks). Possibly this could indicate that the amount of time HIIT is performed per session is more important than the accumulated time over a total training period for improvements in velocity or power output at lactate concentrations between 4 and 10 mmol·L^−1^.

Another aspect that should be mentioned here are the differences in the test protocols when comparing the MART/MACT (25 and 30 s stages with 100 s rest) with a standard incremental protocol (e.g., 3–5 min stages with 20–30 s rest for blood sampling). In this context it is worth noting that in the study of Stöggl and Sperlich (2014) the peak velocity/power at 4 mmol·L^−1^ increased in both POL (+8.1%, *P* < 0.01) and HIIT (+5.6%, *P* < 0.05). Therefore, performance changes at lactate thresholds cannot be directly transferred among different test protocols. This might also be attributed toward the different energy system contributions between the MART/MACT vs. a standard incremental test protocol.

### Limitations, perspectives, and practical applications

One limitation of the current study can be seen in the mix between test modalities applied across the participants by using running or cycling tests specific to their preferred training exercise (e.g., cyclist vs. runner). However, because it is not an easy task to recruit large numbers of well-trained to elite athletes for such an experiment, various types of endurance athletes were included. Furthermore, although the MART was shown to be associated with neuromuscular factors/characteristics (Paavolainen et al., 1994, 1999a,c; Nummela et al., 2006) no specific parameters about effects on neuromuscular components were measured in the current study. Therefore, only indirect conclusions from MART performance changes toward neuromuscular components can be drawn.

Future research about long-term effects of different training intensity distributions in well-trained athletes on aerobic and anaerobic key components of performance is warranted. Still the measurement of HRR in between HIIT bouts, or when using the MART for diagnostics as in the current study, could be a practical tool to track physiological adaptations. In addition, the transfer of these enhanced capacities toward real competition situations has still to be proven.

## Conclusion

In this study of elite athletes performing HIIT, POL, or mainly HVLIT over a period of 9 weeks, only the HIIT group achieved significant improvements (6.4%) in peak performance during the MART/MACT, while, unexpectedly, in no group the performance at the established lactate concentrations (4, 6, 10 mmol·L^−1^) was changed. Acute HRR was improved only in the HIIT (11.2%) and POL (7.9%) group with no change in the HVLIT oriented control group. Therefore, it might be concluded that only a training regime that includes a significant amount of HIIT improves the neuromuscular characteristics, anaerobic power, and the acute HRR in well-trained endurance athletes. A training regime that followed more a HVLIT oriented model had no effect on any performance outcomes. Practically, if HIIT is incorporated during pre-race preparation, i.e., tapering HRR could provide a useful tool for monitoring adaptions related to anaerobic power and physiological response. These findings shed new light into the cardiovascular, central nervous and anaerobic adaptations in response to training regimes with different training intensity distributions and should be of special interest in sports with high intensity intermittent character (e.g., game sports like soccer, ice hockey, and handball) with a substantial anaerobic energy contribution. Also, the results might be of interest for endurance athletes competing in sports using a masstart that involves repetitive high intensity elements that are decisive for the race outcome (e.g., fast accelerations during the start, sprint attacks, and finish spurt).

## Author contributions

Conception and design of the experiments: TS, Performance of the experiments: TS, and GB. Data analysis: TS, and GB. Preparation of the manuscript: TS, and GB. Both authors read and approved the final manuscript.

### Conflict of interest statement

The authors declare that the research was conducted in the absence of any commercial or financial relationships that could be construed as a potential conflict of interest.
